# Diminished secretion and function of IL‐29 is associated with impaired IFN‐α response of neonatal plasmacytoid dendritic cells

**DOI:** 10.1002/JLB.4A0518-189R

**Published:** 2019-06-18

**Authors:** Lukas Wisgrill, Isabelle Wessely, Antonia Netzl, Linda Pummer, Kambis Sadeghi, Andreas Spittler, Angelika Berger, Elisabeth Förster‐Waldl

**Affiliations:** ^1^ Department of Pediatrics and Adolescent Medicine Division of Neonatology Pediatric Intensive Care & Neuropediatrics Medical University of Vienna Vienna Austria; ^2^ Department of Surgery & Core Facility Flow Cytometry Medical University of Vienna Vienna Austria; ^3^ Center for Congenital Immunodeficiencies Medical University of Vienna Vienna Austria

**Keywords:** interleukin‐29, neonatal Immunity, plasmacytoid dendritic cells, toll‐like receptor, type III interferons

## Abstract

Plasmacytoid dendritic cells (pDCs) are key players in the antiviral immune response and type III IFNs such as IL‐29 appear to play a pivotal role in pDC function. Pronounced susceptibility to viral infections in neonates is partly resulting from diminished antiviral immune mechanisms. Accordingly, the aim of the present study was to investigate the impact of IL‐29 in the altered immune response of neonatal pDCs. PBMCs of adult and term newborns were stimulated with CpG‐ODN2216 in the presence or absence of IL‐29 and assessed for IFN‐α production, downstream‐signaling, and activation marker expression. A significantly lower IL‐29 production after TLR9‐specific stimulation was demonstrated in neonatal pDCs. IL‐29 enhanced the IFN‐α production of pDCs in adults compared to newborns. Newborn pDCs displayed a significantly lower surface expression of IL‐10 and IL‐28Rα receptor resulting in diminished STAT1 and IRF7 activation. Interestingly, concomitant stimulation with CpG‐ODN2216/IL‐29 had no impact on the expression of surface activation and maturation markers of pDCs in neither population. The diminished antiviral immune response of neonatal pDCs is associated with reduced production and cellular responses toward IL‐29. Potential therapeutic agents enhancing the IL‐29 response in neonatal pDCs possibly augment viral protection in newborns.

## Graphical abstract

Reduced IL‐29 secretion and responsiveness of neonatal plasmacytoid dendritic cells mainly contributes to reduced IFNα response against viral components.

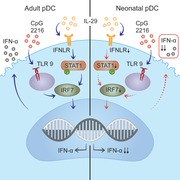

**Abbreviations**
n.s.: not significant
pDC: plasmacytoid dendritic cell

## 1 INTRODUCTION

The susceptibility to infections of newborn infants partly results from a hyporesponsiveness of innate immune responses against bacterial and viral antigens. This “immaturity” of neonatal immune responses is associated with neonatal morbidity and mortality.^1^ In recent years, novel insights in immunological impairments of the neonatal immune system have been described, revealing diminished defending responses against infectious triggers.^2^, ^3^, ^4^

Plasmacytoid dendritic cells (pDCs) are rare peripheral blood cells, however, this small population of cells is a crucial player in the antiviral innate immune response, due to their ability to produce 100 times more type I IFNs than other cell types in response to viral antigens. Although neonatal pDCs show a gestational‐age dependent maturation of early antiviral immune responses after TLR engagement, they still display reduced responses when compared to healthy adults.^4^, ^5^ During the last decade, type III IFNs have been described as new IFN family members adding additional concepts to the complex immunological network of antiviral immune responses. The type III IFN family consists of IL‐29 (IFN‐λ1), IL‐28A (IFN‐λ2) as well as IL‐28B (IFN‐λ3) and was demonstrated to possess potent antiviral activity. Despite the similarity to type I IFNs, type III IFNs signal through distinct receptors. The type III IFN receptor is composed of the IL‐28 receptor α (IL‐28Rα)‐ and the IL‐10 receptor 2 (IL‐10R2)‐chain.^6^ Whereas the IL‐10R2 chain is ubiquitously expressed, the IL‐28Rα receptor appears to be more selectively expressed on immune cells such as pDCs, B cells, or **Mϕ**s.^7^ Both receptor complexes share similar downstream‐signaling pathways including JAK/STAT and IRF proteins. Tyrosine kinase 2 (TYK2) is an important signaling molecule to mount a functional IFN‐α response.^8^ Interestingly, type III IFN signaling and function was not influenced in a patient with TYK2‐deficiency.^9^

Up to date, few studies investigated the physiological and immunomodulatory effect of type III IFNs, especially of IL‐29, on immune cells. IL‐29 appears to augment the antiviral immune response in human peripheral blood and exert different immunomodulatory effects on pDCs.^10^, ^11^, ^12^ To date, data in respect to type III IFNs in neonatal immunity are missing. Detailed knowledge on respective mechanisms and regulatory circuits could open up new concepts of therapeutic or prophylactic immunomodulation upon clinical necessity in a state of immunological impairment. Accordingly, the aim of our study was to delineate the role of type III IFNs in the neonatal early antiviral immune response and, furthermore, to investigate the immunomodulatory properties of IL‐29 on adult and neonatal pDCs.

## 2 MATERIALS AND METHODS

### 2.1 Donors

Blood samples from healthy adult volunteers (age 18–40 years) were obtained by puncture of an antecubital vein after informed consent. Umbilical cord blood was obtained after cesarean section from healthy term newborns (38–42 weeks of gestation). Written informed consent from the mother was obtained prior to birth. The study protocol was approved by the ethics committee of the Medical University of Vienna (1989/2015).

### 2.2 Cell culture

PBMCs were obtained from adult peripheral blood and term newborn cord blood (CBMCs) by Ficoll Hypaque (Amersham Bioscience, Uppsala, SWE) density gradient centrifugation. For better understanding of the manuscript, we use the term PBMCs for both, PBMCs and cord blood mononuclear cells. Fresh PBMCs were directly analyzed by flow cytometry or cultured for further experiments. For stimulation assays, 2 × 10^6^ PBMCs were cultured in 12‐well plates in 2 ml RPMI‐1640 supplemented with 10% FCS (both Thermo Fisher Scientific, Waltham, MA) and 10 ng/ml IL‐3 (Peprotech, Rocky Hill, NJ). PBMCs were treated with vector (PBS, Sigma–Aldrich, St. Louis, MO), IL‐29 (Peprotech), IFN‐α (Peprotech), CpG ODN2216 (InvivoGen, San Diego, CA), or HSA (Sigma–Aldrich) alone or in combination.

### 2.3 Surface and intracellular TLR9 staining of pDCs

PBMCs were stimulated with PBS (negative control) or CpG (positive control) or CpG combined with IL‐29 or HSA (protein control) for 8 h at 37°C. Cells were harvested, washed twice with staining buffer (PBS + 1% FCS), incubated for 5 min with Fc blocking reagent (Miltenyi Biotec, Bergisch Gladbach, GER), and stained for 20 min at 4°C with following anti‐human antibodies: BDCA2 APC (AC144, Miltenyi Biotec), CD123 PerCP‐Cy5.5 (3.9), CD40 FITC (5C3), CD3/CD14/CD16/CD19/CD20/CD56 eFluor450 (Lineage‐marker), ICOS‐L PE (MIH‐12), CCR7 PE‐Cy7 (3D12, all from Thermo Fisher Scientific), HLA‐DR V500 (G46‐6; BD Biosciences), IL‐10R APC (90220), IFNLR1 PE (601106, both from R&D Systems, Minneapolis, MA), IFNAR1 PE (85228, Thermo Fisher), and IFNAR2 APC (REA124, Miltenyi Biotec). Cells were washed, fixed with 4% paraformaldehyde, and immediately analyzed on a LSRII flow cytometer (BD Biosciences). TLR9 staining (TLR9‐PE (eB72‐1665), Thermo Fisher Scientific) was performed on fixed and permeabilized cells using the Intracellular Fixation & Permeabilization Buffer Set (Thermo Fisher Scientific).

### 2.4 Cytokine measurement in cell culture supernatant

PBMCs were stimulated as described above for 24 h at 37°C. Cell free supernatant was obtained by 2 centrifugations steps at 10,000 × *g* for 10 min at 4°C. Supernatant was aliquoted and frozen at –80°C until analysis. IFN‐α (Thermo Fisher Scientific) and IL‐29 (R&D Systems) were assessed using an ELISA according to the manufacturer's protocol.

### 2.5 Intracellular IFN‐α staining

PBMCs were stimulated as described above for 8 h at 37°C. After 2 h of stimulation, brefeldin A (5 µg/ml, Thermo Fisher Scientific) was added to the cell culture. Cells were harvested, washed twice in staining buffer, and surface stained with BDCA2‐ APC (Miltenyi Biotec) and anti‐CD123 PerCP‐Cy5.5 (Thermo Fisher Scientific) for 20 min at 4°C. Afterward, cells were fixed and permeabilized using the Intracellular Fixation & Permeabilization Buffer Set (Thermo Fisher Scientific). Cells were stained with anti‐IFN‐α FITC (EBI‐10, Thermo Fisher Scientific) for 30 min at 4°C, washed twice with staining buffer, and immediately analyzed by flow cytometry.

### 2.6 Intracellular IRF7 staining

PBMCs were stimulated as described above for 16 h at 37°C. Cells were harvested, washed twice in staining buffer, and surface stained with anti‐CD123 PE, anti‐CD11c Alexa Fluor 700 (3.9), and CD3/CD14/CD16/CD19/CD20/CD56 eFluor450 (Lineage‐marker, all from Thermo Fisher) for 20 min at 4°C. Afterward, cells were fixed and permeabilized using the PerFix‐nc Kit from Beckman Coulter (Marseille, FRA) according to the manufacturer's instructions. Intracellular IFN regulatory factor 7 (IRF7) was stained using the anti‐IRF7 Alexa Fluor 647 (RDP4ND4, Thermo Fisher) for 60 min at room temperature in the dark. Cells were washed twice and immediately analyzed using a Cytoflex LX flow cytometer (Beckman Coulter).

### 2.7 STAT1 signaling and protein expression

PBMCs were stimulated with IL‐29 (500 ng/ml) or IFN‐α (500 ng/ml, both from Peprotech) or left unstimulated for 20 min at 37°C. Cells were harvested, washed with ice‐cold staining buffer, blocked with Fc blocking reagent, and surface stained with BDCA‐2 PE (both from Miltenyi Biotec) and CD123 FITC (Thermo Fisher Scientific) for 20 min at 4°C. Cells were fixed with 4% paraformaldehyde for 10 min and permeabilized with PERM Buffer III (BD Biosciences) according to the manufacturer's protocol and stained for 30 min with anti‐pSTAT1(pY701) Alexa Fluor 647 (4a, BD Biosciences). Cells were washed twice with staining buffer and immediately analyzed by flow cytometry. For total STAT1 protein expression, unstimulated PBMCs were analyzed using the total STAT1 ELISA kit (Abcam, Cambridge, UK) according to the manufacturer's instructions.

### 2.8 Fluorescence cell sorting and RNA extraction

For transcriptional analysis, pDCs were purified by FACS using a MoFlo Astrios flow cytometer cell sorter (Beckman Coulter). PBMCs were left untreated or stimulated with CpG alone or in combination with IL‐29 for 3.5 h at 37°C. Cells were harvested and stained with anti‐lineage (CD3/CD14/CD19/CD20/CD56), anti‐CD123, and anti‐BDCA2 for 20 min at 4°C. A minimum of 1000 lineage^−^/CD123^+^/BDCA2^+^ cells were sorted in sterile PCR tubes. Total RNA was extracted using using the RNeasy Plus Mini Kit (Qiagen, Venlo, NL). RNA quantity and quality were assessed using the Agilent Bioanalyzer RNA 6000 nano kit (Agilent Technologies, Waldbronn, GER).

### 2.9 TaqMan quantitative RT‐PCR

The ABI PRISM 7500HT Sequence Detection System (Applied Biosystems, Foster City, CA, USA) and One‐Step RT‐qPCR kit (Bio‐Rad, Hercules, CA, USA) was used for quantitative RT‐PCR analysis. Primer‐probes sets for IFNA FAM and GAPDH VIC were obtained predesigned from Applied Biosystems and tested for primer efficacy (gene expression assays Hs00265051_s1). Multiplex amplification was carried out in a total volume of 20 µl for 40 cycles of 3 s at 95°C and 30 s at 60°C. Initial denaturation was performed for 3 min at 95°C. Target gene expression was normalized to GAPDH housekeeping gene expression. Normalized target gene expression was analyzed by the comparative ΔΔCT method and calculated as x‐fold expression.

### 2.10 Statistical analysis

Statistical analysis was performed with R 3.5.1. Shapiro‐Wilk test was performed to prove normal distribution and Levene test was applied to verify the homogeneity of variance. Data were then analyzed using 1‐way ANOVA in accordance with Tukey. Data that were not normally distributed were analyzed using Kruskal‐Wallis test. Flow cytometry data analysis was carried out with FlowJo X (FlowJo LLC, Ashland, Oregon). A *P*‐value ≤ 0.05 was considered as statistically significant.

## 3 RESULTS

### 3.1 Concomitant TLR9 and type I or III IFN stimulation differentially activate adult and term PBMCs

**Figure 1:**
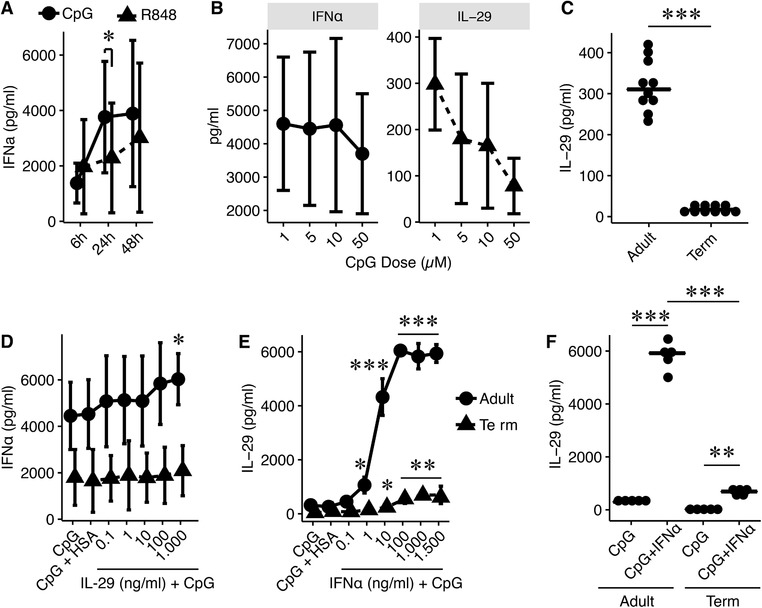
**Concomitant TLR9 and type I or III interferon stimulation differentially activate adult and term PBMCs**. (**A**) Time‐kinetic experiment with adult PBMCs (*n* = 5) stimulating with R848 (TLR7/8 agonist; 100 ng/ml; black triangles) and CpG2216 (TLR9 agonist; 10 µM; black dots). (**B**) Adult PBMCs (*n* = 5) were stimulated for 24 h with increasing doses of CpG2216 and the secretion of IFN‐α (left) and IL‐29 (right) was determined by ELISA. (**C**) IL‐29 release after stimulation of adult and term PBMCs (both groups *n* = 10) for 24 h with CpG2216. (**D**) Dose‐response experiment with increasing dosage of IL‐29 was conducted to determine the effect of IFN‐α release on CpG2216 stimulated adult (*n* = 6; black dots) and term (*n* = 5; black triangles) PBMC cultures. (**E**) Dose‐response experiment with increasing dosages of IFN‐α to assess the impact on IL‐29 release in adult (*n* = 5; black dots) and term (*n* = 5; black triangles) PBMCs after 24 h stimulation. (**F**) Production of IL‐29 in adult probands (*n* = 5) and term newborns (*n* = 5) after stimulation with CpG2216 and 1000 ng/ml IFN‐α. Cytokine levels in supernatants were assessed by ELISA. Data are shown as mean ± sd or as mean alone. Normal distributed data were analyzed using 1‐way ANOVA in accordance with Tukey and not‐normal distributed data were analyzed using Kruskal‐Wallis test or Student's *t*‐test. ^*^ *P* < 0.05; ^**^ *P* < 0.01; ^***^ *P* < 0.001; HSA, human serum albumin

Initially, we investigated the potential of different TLR agonists (Poly(I:C) ‐ TLR3, R848 ‐ TLR7/8; CpG2006 ‐ TLR9; CpG2216 ‐TLR9) to induce IFN‐α production of adult PBMCs. Time‐kinetics revealed a maximum IFN‐α secretion at 24 h after stimulation with CpG2216. R848 induced a significantly lower production of IFN‐α after 24‐h stimulation compared to CpG2216 (Fig. 1A); poly(I:C) as well as CpG2006 had no effect on the IFN‐α production (data not shown). Thus, we chose CpG2216 as optimal stimulus and conducted as next investigational step a dose‐kinetic response. Increasing concentrations up to 50 µM CpG2216 resulted in comparable IFN‐α levels. In case of IL‐29 secretion, stimulation with 1 µM CpG2216 resulted in a significantly higher production of IL‐29 compared to 10 and 50 µM dosages (Fig. 1B), leaving 1 µM CpG2216 as optimal concentration for further experiments. TLR9 specific stimulation led to a significantly higher secretion of IL‐29 in adult PBMCs compared to term newborns (Fig. 1C). Interestingly, CpG2216 had no effect on the secretion of IL‐28A and IL28B in our experimental setting (data not shown).

Next, we investigated the immunomodulatory effect of concomitant TLR9 and type I or III IFNs on the secretion of the complementary IFN type. First, we analyzed the dose‐response relation of IL‐29 showing a significant higher IFN‐α secretion in adult PBMCs when simultaneously stimulated with 1000 ng/ml IL‐29. In contrast, term PBMCs did not respond to concurrent IL‐29 stimulation displaying significantly lower IFN‐α levels (Fig. 1D). Subsequently, concomitant stimulation with CpG2216 and increasing IFN‐α dosages resulted in a significant increase of IL‐29 secretion in both groups (Fig. 1E). Adult PBMCs demonstrated a significantly higher IL‐29 production after stimulation with CpG2216 and 1000 ng/ml IFN‐α compared to term newborns (Fig. 1F).

### 3.2 Newborn pDCs exhibit diminished response against IL‐29

**Figure 2:**
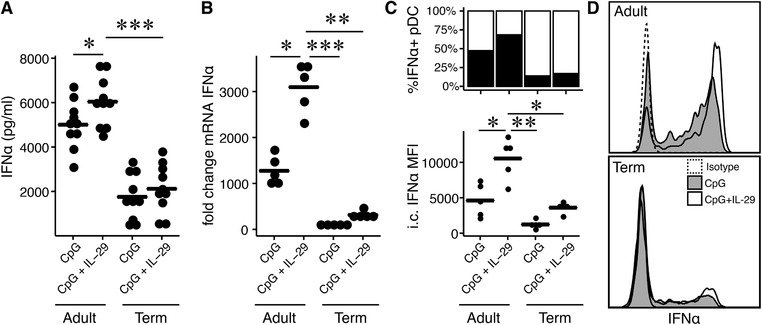
**Production of IFN‐α in plasmacytoid dendritic cells (pDCs)**. (**A**) Secretion of IFN‐α in adult probands (*n* = 10) and term newborns (*n* = 10) after stimulation with CpG2216 alone or in combination with IL‐29. Supernatants were analyzed by ELISA. (**B**) PDCs were sorted by FACS and the IFN‐α mRNA expression upon CpG2216 stimulation alone or in combination with IL‐29 was assessed by RT‐PCR of healthy adults (*n* = 5) and term newborns (*n* = 5). IFN‐α mRNA was normalized to 18s RNA and IFNα mRNA expression was compared between CpG2216‐stimulated and untreated cells. Values are shown as fold change in relation to unstimulated control. (**C**) PDCs of adults (*n* = 5) and term newborns (*n* = 5) were stimulated with CpG2216 or IL‐29 alone and in combination of both for 8 h and IFN‐α production was assessed by intracellular staining via flow cytometry (lower panel). The percentage of IFN‐α positive pDCs (black bars) are shown in the upper panel. (**D**) Representative histograms of 6 independent experiments of the intracellular levels of IFN‐α in pDCs. The black line in dotplots represents the mean value. Normal distribution was determined with Shapiro‐Wilk test. Normal distributed data was analyzed using 1‐way ANOVA in accordance with Tukey and not‐normal distributed data were analyzed using Kruskal‐Wallis test. ^*^ *P* < 0.05; ^**^ *P* < 0.01; ^***^ *P* < 0.001; MFI, mean fluorescence intensity

In the next step, we investigated the impact of simultaneous stimulation with CpG2216 and IL‐29 on the single cell level in newborns and adults. Stimulating PBMCs simultaneously with CpG2216 and IL‐29 resulted in significantly higher IFN‐α levels in adult probands compared to CpG2216 alone. On the contrary, IFN‐α secretion in newborns was significantly diminished after stimulation with CpG2216 alone or in combination with CpG2216/IL‐29 compared to healthy adult controls (Fig. 2A). In addition, we investigated whether IL‐29 affects IFN‐α expression on a transcriptional level. Adult pDCs showed a significantly higher expression of IFN‐α mRNA after stimulation with CpG2216/IL‐29 compared to CpG2216 alone. Furthermore, adult pDCs showed a significantly higher IFN‐α mRNA expression after both stimuli compared to term newborns (Fig. 2B). To validate our culture supernatant results and mRNA experiments, we performed an intracellular IFN‐α staining assay. Accordingly, a significantly higher intracellular concentration of IFN‐α after co‐incubation with IL‐29 and CpG2216 was observed in healthy adult pDCs. However, pDCs from term newborns showed a significantly lower production of IFN‐α after stimulation with CpG2216 alone or in combination with IL‐29/CpG2216 (Fig. 2C lower panel and D), and the amount of IFN‐α increase upon IL‐29 stimulation was not significant in comparison to CpG alone. Interestingly, a higher fraction as well as higher IFN‐α production per pDC was observed in adult probands and with no effect in the newborn group, which is illustrated in the upper panel of Fig. 2C.

### 3.3 Downstream signaling of IL‐29 is decreased in neonatal pDCs

**Figure 3:**
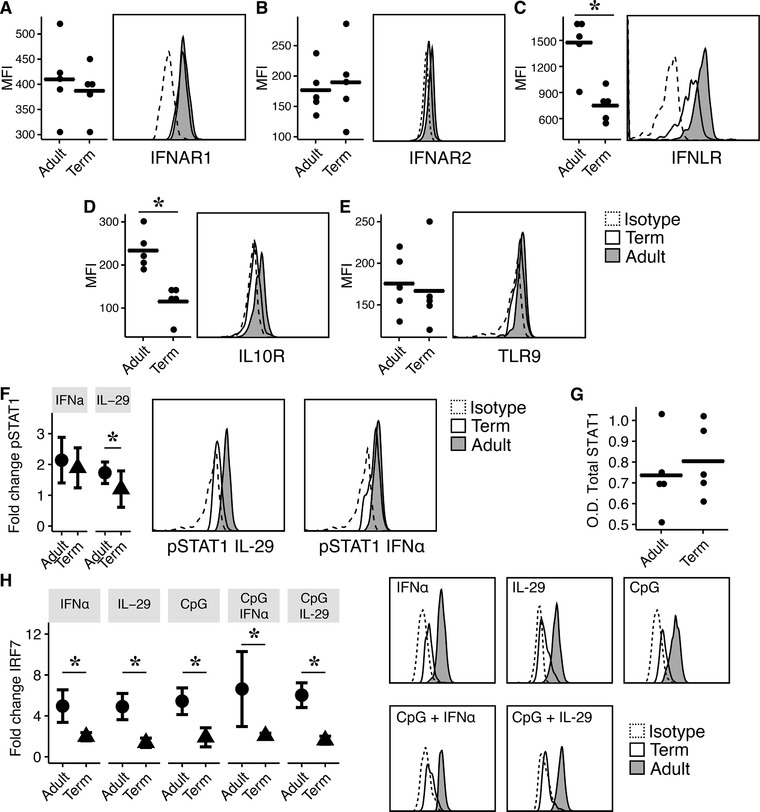
**Expression of surface receptors and associated downstream molecules of type I and type III IFNs on plasmacytoid dendritic cells (pDCs)**. Surface expression of (**A**) IFNAR1, (**B**) IFNAR2, (**C**) IFNLR, (**D**) IL10R as well as intracellular expression of (**E**) TLR9 was evaluated by flow cytometry in healthy adults (*n* = 5) and term newborns (*n* = 5). Representative histograms of 12 independent experiments are depicted (**F**) Phosphorylation level of signal transducer and activator of transcription 1 (STAT1) in pDCs of adults (*n* = 5; black bars) and term newborns (*n* = 5; white bars) after stimulation with IFN‐α (500 ng/ml) and IL‐29 (500 ng/ml) for 20 min. Signaling was analyzed using flow cytometry. Representative histograms of 5 independent flow cytometry experiments of the phosphorylation levels of STAT1 in pDCs after activation with IFN‐α and IL‐29 are shown. (**G**) Protein expression of STAT1 in adult (*n* = 5) and term (*n* = 5) PBMC cell lysate determined by semiquantitative ELISA. (**H**) Expression levels of IRF7 in adult (*n* = 4) and term (*n* = 4) pDCs after 16 h stimulation with IFN‐α, IL‐29, or CpG2216 alone or in combination. IRF7 levels were determined by flow cytometry. Representative histograms from 1 of 6 independent experiments are shown. Data are shown as mean ± sd or as mean alone. Normal distribution was determined with Shapiro‐Wilk test. Normal distributed data were analyzed using 1‐way ANOVA in accordance with Tukey or Student *t*‐test and not‐normal distributed data was analyzed using Mann‐Whitney or Kruskal‐Wallis test. ^*^ *P* < 0.05; MFI, mean fluorescence intensity; OD, optical density

To determine the mechanism behind the diminished functional response against IL‐29 of newborn pDCs, we investigated the expression of surface receptors and associated signaling molecules of type I and III IFNs pathways. First, we examined the expression of the involved receptors for IFN‐α (IFNAR1/IFNAR2), IL‐29 (IFNLR/IL10R), and CpG2216 (TLR9) recognition. Adult pDCs expressed significantly higher levels of IFNLR and IL10R compared to term newborns (Fig. 3C and D). IFNAR1, IFNAR2, and TLR9 expression was similar between healthy adults and term newborns (Fig. 3A, B, and E). Next, we investigated the phosphorylation levels of STAT1 of pDCs after stimulation with IFN‐α or IL‐29. We detected significantly lower levels of phosphorylated STAT1 after IL‐29 challenge in term newborns compared to adults. Stimulation with IFN‐α exhibited comparable phosphorylation levels of STAT1 in both age groups (Fig. 3F). The overall expression of STAT1 in adult and newborn PBMCs showed no difference in both groups (Fig. 3G).

Next, we investigated the expression levels of the transcription factor IRF7 in adult and neonatal pDCs. Adult pDCs showed a significant up‐regulation of IRF7 after CpG, type I and III IFNs as well as combined stimulation compared to term infants. In infants, stimulation with the indicated stimuli had no significant effect on IRF7 up‐regulation (Fig. 3H). Unfortunately, we were not able to detect phosphorylated IRF7 via flow cytometry.

### 3.4 Combined stimulation with CpG2216 and IL‐29 had no impact on the expression of surface activation marker

**Figure 4:**
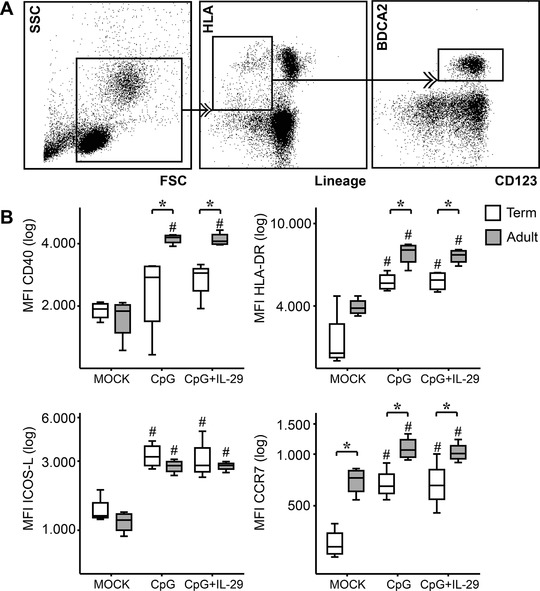
**Gating strategy and surface activation and maturation marker expression on plasmacytoid dendritic cells (pDCs)**. (**A**) Peripheral blood pDCs were identified by the lack of lineage‐marker and the expression of HLA‐DR, CD123, and BDCA2. (**B**) Surface expression of CD40, HLA‐DR, ICOS‐L, and CCR7 was evaluated by flow cytometry in healthy adults (*n* = 4; gray boxes) and term newborns (*n* = 4; white boxes) after stimulation with CpG2216 alone or in combination with IL‐29. Boxes display the 25th and 75th percentiles and error bars indicate the 5th and the 95th percentiles. Median values are represented by the box middle line. Normal distribution was determined with Shapiro‐Wilk test. Normal distributed data were analyzed using 1‐way ANOVA in accordance with Tukey and not‐normal distributed data were analyzed using Kruskal‐Wallis test. ^*^ *P* < 0.05; ^#^ *P* < 0.05 compared to respective MOCK control

Next, we aimed to investigate the effect of concomitant stimulation with CpG2216 and IL‐29 on the expression of surface activation and maturation marker of pDCs. Upon CpG2216 stimulation, adult and neonatal pDCs displayed a significantly higher expression of HLA‐DR, ICOS‐L, and CCR7 compared to unstimulated controls. CD40 was not significantly up‐regulated after CpG2216 stimulation in term neonates. Adult pDCs exhibited significantly higher expression levels of CD40, HLA‐DR, and CCR7 after CpG2216 engagement compared to neonates. Overall, the concomitant stimulation with CpG2216 and IL‐29 had no influence on the expression pattern of CD40, HLA‐DR, ICOS‐L, and CCR7 in both study groups. (Fig. 4).

## 4 DISCUSSION

IL‐29 exerts a variety of immunomodulatory effects on various cell types.^10^, ^13^, ^14^, ^15^ In recent years, several reports have shed light onto the type III IFN system and its role during viral infections such as airway‐inflammation^16^ or hepatitis.^17^ Reduced production of IFN‐α in response to RSV infection is associated with a significant increase in the frequency of upper respiratory infections and pneumonia.^18^ Important cellular players of innate immune responses fighting viral pathogens are pDCs. Although low in number in circulating blood, pDCs are the major producers of IFN‐α after viral pathogen encounter.^19^ Recently, several reports described the immunomodulatory effect of IL‐29 on the enhancement of IFN‐α production in human pDCs.^12^, ^15^, ^20^ In our previous studies, we had demonstrated that pDCs from term and preterm neonates exhibit diminished IFN‐α production and decreased expression of activation markers after TLR‐specific triggers, which is thought to contribute to the susceptibility toward viral infections in neonates.^3^ ^4^ Now, for the first time, we analyzed the production as well as the immunomodulatory effects of IL‐29 on adult and neonatal PBMC and pDCs.

First, we determined the role of IL‐29 in induction of IFN‐α upon TLR‐specific engagement. In addition to pDCs, BDCA3^+^ myeloid dendritic cells (mDCs) are described to be a major IL‐29 producer in peripheral blood after TLR3‐specific stimulation.^21^ Therefore, besides TLR7/8 and TLR9‐agonists, we included poly(I:C) as TLR3‐specific agent. In our experimental setting, we could only detect a robust IL‐29 production in adult PBMCs—but not in neonatal PBMCs—upon stimulation with CpG2216. Our observations stand in line with previous published results, showing a diminished IL‐29 response of neonatal mDCs in‐vitro after infection with human cytomegalovirus.^22^ These lower levels of IFNs cannot be explained by different cell counts of pDCs or BDCA3^+^ mDCs as shown in our previous study.^4^ Thus, this impaired IL‐29 secretion might be an intrinsic functional deficiency of the neonatal innate immunity.

IL‐29 appears to exhibit direct immunomodulatory effects on pDCs, as mentioned by engagement of a receptor being built of a heterodimer of the IL28Rα‐ and the IL10R2‐chain. First described in mice, knock out of the type III IFN receptor IL‐28Rα resulted in a diminished antiviral‐like immune response after encounter with TLR3 or TLR9 agonists.^23^ In humans, pDCs display a high constitutive surface expression of IL‐28Rα and, additionally, was shown to up‐regulate the receptor after TLR‐specific stimulation.^10^ Thus, activation of pDCs seem to prone the cell to respond more effectively to paracrine/autocrine secreted IL‐29. In consequence, we hypothesized that the functional deficiency of the type III IFN system might be due to altered surface receptor expression. Neonates displayed a significantly lower expression of IL‐28Rα resulting in significantly lower phosphorylation of STAT1 and expression of IRF7 after stimulation with IL‐29. However, the activation of STAT1 was similar in neonates and healthy adults in pDCs after IFN‐α stimulation. Hence, neonates not only display an impaired IL‐29 production after TLR‐specific activation, but furthermore show a diminished type III IFN response upon IL‐29 encounter of pDCs. Our findings are underlined by the study of Danis et al.^24^ showing an impaired nuclear translocation of IRF7 in neonatal pDCs. Facing the fact that innate immune responses train the adaptive immunity in order to promptly and efficiently react on specific antigen encounter, we clearly postulate that diminished innate immune responses of neonates have a relevant impact on the antiviral immune response of this early age infants.

Recent studies implicate that IL‐29 enhances the IFN‐α response of human mononuclear cells indicating a potential therapeutic use boosting the neonatal immune responses.^11^, ^20^ As a consequence, we investigated the immunostimulatory properties of recombinant IL‐29 to augment the IFN‐α response of term newborn and adult pDCs. Strikingly, IL‐29 showed a dose‐dependent increase of IFN‐α secretion in adult pDCs but not in term neonates. Using high doses up to 1000 ng/ml, we tried to mimic high IL‐29 concentration of the pDCs micro‐milieu in our PBMC cell culture as this cell population is capable of secreting high amounts of IL‐29 after CpG stimulation.^10^ To our knowledge, we here presented for the first time, that the reduced IL‐28Rα expression and concomitant signaling via STAT1 and IRF7 results in diminished functional IL‐29 response in neonatal pDCs revealing a novel cellular paracrine mechanism behind impaired antiviral immune responses of neonates. Furthermore, the secretion of IL‐29 in neonates was significantly enhanced via stimulation of IFN‐α suggesting that the dampened production of IL‐29 might be due to reduced primary IFN‐α secretion as well as diminished IL‐29 responsiveness.

Our last investigational step was to investigate the notion that IL‐29 may exhibit its effects by influencing the expression profiles of activation and maturation markers of pDCs. Correspondingly, concomitant stimulation with CpG2216 and IL‐29 did alter the expression of T cell co‐stimulatory molecules and homing receptors of pDCs possibly paving the way for the initiation of an adaptive immune response. Thus, IL‐29 mediates its immunomodulatory antiviral effects on pDCs by enhancing the primary cytokine response, but by altering expression profiles of activation and homing markers after TLR9‐specific engagement.

The impaired ability of neonatal pDCs to initiate an adequate antiviral immune response upon TLR‐specific activation implies clinical and immunological relevance in this infection prone population. Indeed, IL‐29 seems to be a promising candidate for the treatment of acute and chronic viral infections such as hepatitis C or E.^25^, ^26^ However, based on our data, therapeutic approaches targeting IL‐29 might display reduced efficacy in neonates. Megjugorac et al. described that IL‐4 lead to higher secretion of IL‐1 receptor agonist (IL‐1Ra) in monocytes resulting in augmented IL‐29 output of pDCs. Thus, targeting pathways such as IL‐1Ra might be beneficial to augment the IFN‐α response of neonatal pDCs. Neonates seem to produce comparable amounts of IL‐1Ra during bacterial infections compared to adults.^27^ The role of IL‐1Ra in neonatal viral infections and the impact on the IFN‐α response will need to be elucidated in more detail in the future.

We conclude that not only the diminished IL‐29 production but moreover lack of efficient IL‐29 responsiveness is associated with the impaired capability of neonatal pDCs to secrete IFN‐α after TLR9‐specific stimulation. This deprived loop of IFN‐α associated immunological answers leaves newborns, specifically preterm newborns, susceptible to severe viral infections. A deeper understanding of the ontogeny of the human immune system provides relevant insights into the complex cellular mechanistics underlying newborn antiviral immune responses. Detailing the multiple players and their dynamics is essential when facing the challenge to identify potential immunomodulating targets overcoming innate immune immaturity.

## DISCLOSURE

The authors declare no commercial or financial conflict of interest.

## References

[1] Lawn JE , Kerber K , Enweronu‐Laryea C , Cousens S . 3.6 million neonatal deaths–what is progressing and what is not? Semin Perinatol. 2010;34:371‐386. 21094412 10.1053/j.semperi.2010.09.011

[2] Wynn JL , Levy O . Role of innate host defenses in susceptibility to early‐onset neonatal sepsis. Clin Perinatol. 2010;37:307‐337. 20569810 10.1016/j.clp.2010.04.001 PMC2891962

[3] Schuller S , Wisgrill L , Sadeghi K , et al. The TLR‐specific adjuvants R‐848 and CpG‐B endorse the immunological reaction of neonatal antigen presenting cells. Pediatr Res. 2016. 10.1038/pr.2016.71 27057737

[4] Schuller SS , Sadeghi K , Wisgrill L , et al. Preterm neonates display altered plasmacytoid dendritic cell function and morphology. J Leukoc Biol. 2013;93:781‐788. 23401600 10.1189/jlb.1011525

[5] De Wit D , Olislagers V , Goriely S , et al. Blood plasmacytoid dendritic cell responses to CpG oligodeoxynucleotides are impaired in human newborns. Blood. 2004;103:1030‐1032. 14504106 10.1182/blood-2003-04-1216

[6] Kotenko SV , Gallagher G , Baurin VV , et al. IFN‐lambdas mediate antiviral protection through a distinct class II cytokine receptor complex. Nat Immunol. 2003;4:69‐77. 12483210 10.1038/ni875

[7] Sheppard P , Kindsvogel W , Xu W , et al. IL‐28, IL‐29 and their class II cytokine receptor IL‐28R. Nat Immunol. 2003;4:63‐68. 12469119 10.1038/ni873

[8] Prchal‐Murphy M , Semper C , Lassnig C , et al. TYK2 kinase activity is required for functional type I interferon responses in vivo. PLoS One. 2012;7:e39141. 22723949 10.1371/journal.pone.0039141 PMC3377589

[9] Fuchs S , Kaiser‐Labusch P , Bank J , et al. Tyrosine kinase 2 is not limiting human antiviral type III interferon responses. Eur J Immunol. 2016;46:2639‐2649. 27615517 10.1002/eji.201646519

[10] Megjugorac NJ , Gallagher GE , Gallagher G . Modulation of human plasmacytoid DC function by IFN‐lambda1 (IL‐29). J Leukoc Biol. 2009;86:1359‐1363. 19759281 10.1189/jlb.0509347

[11] Cho CH , Yoon SY , Lee CK , Lim CS , Cho Y . Effect of interleukin‐29 on interferon‐alpha secretion by peripheral blood mononuclear cells. Cell J. 2015;16:528‐537. 25685743 10.22074/cellj.2015.497 PMC4297491

[12] Megjugorac NJ , Gallagher GE , Gallagher G . IL‐4 enhances IFN‐lambda1 (IL‐29) production by plasmacytoid DCs via monocyte secretion of IL‐1Ra. Blood. 2010;115:4185‐4190. 20233967 10.1182/blood-2009-09-246157

[13] de Groen RA , Groothuismink ZM , Liu BS , Boonstra A . IFN‐lambda is able to augment TLR‐mediated activation and subsequent function of primary human B cells. J Leukoc Biol. 2015;98:623‐630. 26130701 10.1189/jlb.3A0215-041RR

[14] Dai J , Megjugorac NJ , Gallagher GE , Yu RY , Gallagher G . IFN‐lambda1 (IL‐29) inhibits GATA3 expression and suppresses Th2 responses in human naive and memory T cells. Blood. 2009;113:5829‐5838. 19346497 10.1182/blood-2008-09-179507

[15] Jordan WJ , Eskdale J , Srinivas S , et al. Human interferon lambda‐1 (IFN‐lambda1/IL‐29) modulates the Th1/Th2 response. Genes Immun. 2007;8:254‐261. 17361203 10.1038/sj.gene.6364382

[16] Selvaggi C , Pierangeli A , Fabiani M , et al. Interferon lambda 1–3 expression in infants hospitalized for RSV or HRV associated bronchiolitis. J Infect. 2014;68:467‐477. 24389019 10.1016/j.jinf.2013.12.010 PMC7172705

[17] Park H , Serti E , Eke O , et al. IL‐29 is the dominant type III interferon produced by hepatocytes during acute hepatitis C virus infection. Hepatology. 2012;56:2060‐2070. 22706965 10.1002/hep.25897 PMC3581145

[18] Sumino K , Tucker J , Shahab M , et al. Antiviral IFN‐gamma responses of monocytes at birth predict respiratory tract illness in the first year of life. J Allergy Clin Immunol. 2012;129:1267‐1273. e1. 22460071 10.1016/j.jaci.2012.02.033 PMC3340511

[19] Fitzgerald‐Bocarsly P . Natural interferon‐alpha producing cells: the plasmacytoid dendritic cells. Biotechniques. 2002;16–20:24‐29. 22. 12395924

[20] Yin Z , Dai J , Deng J , et al. Type III IFNs are produced by and stimulate human plasmacytoid dendritic cells. J Immunol. 2012;189:2735‐2745. 22891284 10.4049/jimmunol.1102038 PMC3579503

[21] Lauterbach H , Bathke B , Gilles S , et al. Mouse CD8alpha+ DCs and human BDCA3+ DCs are major producers of IFN‐lambda in response to poly IC. J Exp Med. 2010;207:2703‐2717. 20975040 10.1084/jem.20092720 PMC2989774

[22] Renneson J , Dutta B , Goriely S , et al. IL‐12 and type I IFN response of neonatal myeloid DC to human CMV infection. Eur J Immunol. 2009;39:2789‐2799. 19637227 10.1002/eji.200939414

[23] Ank N , Iversen MB , Bartholdy C , et al. An important role for type III interferon (IFN‐lambda/IL‐28) in TLR‐induced antiviral activity. J Immunol. 2008;180:2474‐2485. 18250457 10.4049/jimmunol.180.4.2474

[24] Danis B , George TC , Goriely S , et al. Interferon regulatory factor 7‐mediated responses are defective in cord blood plasmacytoid dendritic cells. Eur J Immunol. 2008;38:507‐517. 18200500 10.1002/eji.200737760

[25] Bruening J , Weigel B , Gerold G . The role of type III interferons in hepatitis C virus infection and therapy. J Immunol Res. 2017;2017:7232361. 28255563 10.1155/2017/7232361 PMC5309426

[26] Donnelly RP , Dickensheets H , O'Brien TR . Interferon‐lambda and therapy for chronic hepatitis C virus infection. Trends Immunol. 2011;32:443‐450. 21820962 10.1016/j.it.2011.07.002 PMC3163738

[27] de Bont ES , de Leij LH , Okken A , Baarsma R , Kimpen JL . Increased plasma concentrations of interleukin‐1 receptor antagonist in neonatal sepsis. Pediatr Res. 1995;37:626‐629. 7603782 10.1203/00006450-199505000-00012

